# Participation of FaTRAB1 Transcription Factor in the Regulation of *FaMADS1* Involved in ABA-Dependent Ripening of Strawberry Fruit

**DOI:** 10.3390/foods12091802

**Published:** 2023-04-26

**Authors:** Wenjing Lu, Xiaopeng Wei, Xueyuan Han, Renchi Chen, Chaogeng Xiao, Xiaojie Zheng, Linchun Mao

**Affiliations:** 1 Institute of Food Science, Zhejiang Academy of Agricultural Sciences, 298 Desheng Road, Hangzhou 310021, China; wenjing_316@163.com (W.L.); xiaochaogeng@163.com (C.X.); 2 Zhejiang Key Laboratory of AgroFood Processing, College of Biosystems Engineering and Food Science, Zhejiang University, Hangzhou 310058, China; weixiaopeng007@163.com (X.W.); 15009256461@163.com (X.H.); crc@zju.edu.cn (R.C.); 3 School of Food and Bioengineering, Zhengzhou University of Light Industry, Zhengzhou 450002, China; 4 School of Life Sciences, Shaoxing University, Shaoxing 312000, China; 5 Department of Agriculture and Biotechnology, Wenzhou Vocational College of Science and Technology, Wenzhou 325006, China; 6 Ningbo Research Institute, Zhejiang University, Ningbo 315100, China

**Keywords:** phytohormone, *Fragaria × ananassa*, fruit ripening, MADS, TRAB

## Abstract

Abscisic acid (ABA) plays a crucial role in regulating the ripening of non-climacteric strawberry fruit. In the present study, ABA was confirmed to promote strawberry ripening and induce the down-regulation of *FaMADS1*. The transient silence of *FaMADS1* in strawberries promoted fruit ripening and induced the content of anthocyanin and soluble pectin but reduced firmness and protopectin through a tobacco rattle virus-induced gene silencing technique. In parallel with the accelerated ripening, the genes were significantly induced in the transiently modified fruit, including anthocyanin-related *PAL6*, *C4H*, *4CL*, *DFR*, and *UFGT*, softening-related *PL* and *XTH*, and aroma-related *QR* and *AAT2.* In addition, the interaction between *FaMADS1* and ABA-related transcription factors was researched. Yeast one-hybrid analysis indicated that the *FaMADS1* promoter could interact with FaABI5-5, FaTRAB1, and FaABI5. Furthermore, dual-luciferase assay suggested that FaTRAB1 could actively bind with the *FaMADS1* promoter, resulting in the decreased expression of *FaMADS1*. In brief, these results suggest that the ABA-dependent ripening of strawberry fruit was probably inhibited through inhibiting *FaMADS1* expression by the active binding of transcript FaTRAB1 with the *FaMADS1* promoter.

## 1. Introduction

Phytohormones strongly dominate transcription factors and genetic regulators to regulate fruit ripening, and the degree of fruit maturity determines fruit quality [1]. As one of the main classical phytohormones, abscisic acid (ABA) plays a crucial role in many physiological processes during plant growth and abiotic stress responses, particularly in fruit ripening [2,3,4]. Studies have revealed that strawberry ripening is controlled by ABA [5,6,7] through the expression of related genes and miRNAs [8,9,10].

ABA functioned through a network of signaling pathways, and numerous signaling components were identified. The most prominent upstream regulators were putative ABA receptors, which perceived ABA signals and then triggered downstream signaling cascades, ultimately inducing physiological responses. In plants, three kinds of ABA receptors have been identified: normal G protein-coupled receptors (GCR) and novel GTP-binding protein receptors (GTG) in the cytoplasmic membrane [11], PYL/PYR/RCAR receptors in the cytosol [12], and ABAR/CHLH receptors in plastid or chloroplast [13]. In normal conditions, the ABA signal was perceived and transferred by central transduction complex PYR/PP2Cs/SnRK2s [14]. Activated SnRK2 switched on ABA signaling by phosphorylating the downstream transcription factors such as AREB/ABF, activating several genes sets [15,16]. Moreover, ABAR could repress the expression of *WRKY40* in response to a high level of ABA signal, thereby releasing the expression of the *ABI5* gene [17]. A primary region leucine zipper (bZIP) factor, TRAB1, could interact with ABREs and has been identified as a true trans-acting factor involved in ABA-inducible transcription, which controlled maturation and dormancy in plant embryos [18,19]. However, the complexity and coordination of the network of ABA signaling pathways were more widespread than previously believed.

MADS-box genes are present in nearly all major eukaryotic groups and are pivotal regulators in several processes during plant vegetative and reproductive development [20]. Previous studies have reported that MADS-box genes might be the downstream targets of ABA signaling to participate in ABA-associated plant development. In barley, two MADS-box genes, *HvOS1* and *HvOS2*, are induced by exogenous ABA, and differences in DNA methylation difference after the application of ABA were suggestive of epigenetic regulation of these genes [20]. MADS-box genes also participated in the ABA-associated flower and fruit development [21,22,23]. During the transition from inflorescence to floral meristems, some MADS-box genes have been confirmed to influence the meristem identify genes, regulating the flowering time [24,25]. A SEP-like gene *MADS7* was cloned in sweet cherry, an indispensable positive regulator of fruit ripening, and exogenous ABA could rescue phenotype defects induced by *PaMADS7* silencing [26]. A C-type MADS-box gene *FaSHP* was induced by ABA in either over-expression or RNAi-mediated down-regulation of strawberry fruit, and over-expression of *FaSHP* promoted fruit transformation from green to pink, whereas down-regulation of *FaSHP* could delay the aforementioned transformation [6]. Strawberry *FaMADS1* belonged to the SEP1/2 clade based on the amino acid sequence and was suppressed by ABA treatment during accelerated ripening [27,28].

To research the interaction between *FaMADS1* and ABA-related transcription factors, strawberry fruit were injected with ABA and a suspension of *A. tumefaciens* cells carrying a construct for *FaMADS1*-RNAi to analyze the function of *FaMADS1*. The activity regulation of the five ABA-related transcription factors (FaABI5-5, FaTRAB1, FaABI5, FaABI5-2, and FaABI5-3) to *FaMADS1* was then identified using yeast one-hybrid analysis and dual-luciferase assay.

## 2. Materials and Methods

### 2.1. Fruit and ABA Injection

Octaploid strawberry (*Fragaria × ananassa* Duch. cv. Akihime) plants were grown in suitable environmental conditions. The plastic greenhouse in Hangzhou, Zhejiang Province, China (30°15′ N 120°10′ E), was used for fruit development with a temperature range of 15–25 °C, relative humidity of 70−90%, and no supplemental light. Strawberry fruit at different stages (SG: small green fruit, LG: large green fruit, DG: de-green fruit, W: white fruit, IR: initial red fruit, PR: partial red fruit, and FR: full red fruit) were collected for analysis [7].

Twenty fruit on the plant at DG stage were injected with 0.1 mL of 1 mM ABA (dissolved in 2% ethanol *v*/*v* total volume) into the receptacle core through the pedicel using a sterile microsyringe. Another twenty fruit were injected with 2% ethanol as the control [29]. At 0 d, samples of the control and ABA treatment were collected immediately after injection, and subsequently, all samples were collected after 3, 6, and 9 d. After sanitizing with 1% sodium hypochlorite solution, all fruit was washed with ddH_2_O, cut into small cubes devoid of achenes with a sterilized blade on a sterile bench, and then rapidly frozen in liquid nitrogen [28].

### 2.2. Determination of Fruit Firmness, Total Anthocyanin, Soluble Solid, Titratable Acid, Protopectin, and Soluble Pectin

The firmness of the fruit was determined using a TA-XT2i Texture Analyzer (Stable Micro Systems Ltd., Surrey, UK). According to the manufacturer, two opposite sides of each fruit were identified as the detection site, and a 5 mm diameter probe was used for testing strawberry fruit. During the experiment, a 5 mm penetration depth and a speed of 1 mm s^−1^ were established, and the resulting value, N, was recorded.

Ten strawberry fruit were longitudinally cut into four identical portions and distributed into four subgroups to determine the contents of total anthocyanin, soluble solid, titratable acid, protopectin, and soluble pectin. The content of total anthocyanin (TAC) was detected using the pH differential method [30]. The content of soluble solid was directly measured using a Pocket Refractometer (PAL-1, Atago Co. Ltd., Tokyo, Japan). The total titratable acid content was determined using the acid–base titration method, according to Kafkas et al. [31]. The contents of soluble solid and titratable acidity were expressed as %. The contents of protopectin and soluble pectin were measured by coloring carbazole/vitriol according to Lei et al. [32].

### 2.3. RNA Extraction and RT-qPCR Analysis

Strawberry RNA was extracted using the cetyltrimethylammonium bromide (CTAB) method [33]. The extraction buffer was prepared before RNA extraction, including 2% CTAB, 2% PVP, 2 mol L^−1^ NaCl, 30 mmol L^−1^ spermidine, 100 mmol L^−1^ Tris-HCl (pH 8.0), 25 mmol L^−1^ EDTA (pH 8.0), chloroform:isoamyl alcohol (24:1), 10 mol L^−1^ LiCl, and 2% *β*-mercaptoethanol, which was added just before use. Furthermore, SSTE was prepared for RNA extraction. The RNA was reverse transcribed using the PrimeScript^TM^ RT reagent Kit with gDNA Eraser (Perfect Real Time) (Takara Biotech, Dalian, China). SYBR^®^PremixEx Taq™ (TliRNaseH Plus) (Takara Biotech, Dalian, China) was used for quantitative PCR. *FaActin* gene (GenBank No.: AB116565.1) was considered a loading control gene. The 2^−ΔΔCT^ method was used to calculate the relative gene expressions.

### 2.4. RNAi Plasmid Construction

VIGS vectors of pTRV1 and pTRV2 were selected as described by Liu et al. [34]. The *FaMADS1* coding sequence (750 bp) was cloned in the vector pTRV2 to obtain the *FaMADS1* down-expression construct. The cDNA was inserted into the vector pTRV2 via *Xba*I and *Eco*RI sites. The recombinant plasmid pTRV2-*FaMADS1* was introduced into *Agrobacterium tumefaciens* (strain EHA105). *Agrobacterium* cells were harvested when the culture reached an OD_600_ of 0.8. The infection method was performed according to Jia et al. [5]. The fruit was gathered when either one of the two groups turned red. The expression of *FaMADS1* in *FaMADS1*-RNAi fruit was examined using the primers *FaMADS1*-F and *FaMADS1*-R (Table S1).

### 2.5. Determination of Fruit Aroma Components

Headspace solid-phase microextraction (HS-SPME) was adopted to prepare the samples, and gas chromatography-mass spectrometry (GC-MS) was used to analyze fruit aroma production as described by Vandendriessche et al. [35]. Pure 2-Octanone was added to each sample at a concentration of 0.00524 g L^−1^ to serve as an internal standard composition. The peak areas of components relating to the internal standard were considered for each peak. All extractions were performed manually using a Supelco fiber holder (Bellefonte, PA, USA), and volatile flavor compounds were extracted using a 50/30 μm DVB/CAR/PDMS fiber. Five grams of fruit puree mixed with 5 mL saturated salt water and 50 μL of internal standard (0.00524 g L^−1^, 2-octanone) were added into a 20 mL headspace vial. Semi-quantitative results are expressed as μg kg^−1^ FW compared with the standard.

### 2.6. Determination of Enzyme Activity

The activities of PAL (phenylalanine ammonia lyase), 4CL (*ρ*-coumarate ligase), C4H (cinnamicacid-4-hydroxylase), DFR (dihydroflavonol 4-reductase), PG (polygalacturonase), PL (pectate lyase), and AAT (alcohol acyltransferase) were detected in the control and *FaMADS1*-RNAi fruit. PAL, C4H, and DFR enzyme activities were measured according to Chen et al. [36]. PG enzyme activity was measured using the protocol of Andrews and Li [37]. PL enzyme activity was detected using the protocol of Mao et al. [38]. AAT enzyme activity was determined using the protocol of Pérez et al. [39].

### 2.7. Subcellular Localization Assay

The open reading frame of *FaMADS1* was cloned as C-terminal fusion in frame with the green fluorescent protein (GFP) gene into the pBI121 vector. The fusion construction and infection methods were performed according to Jia et al. [5] and Lu et al. [40]. The *Agrobacterium* suspension was evenly injected into the back of the leaf attached to the BY-2 tobacco (*Nicotiana tabacum*). After 48 h, at least six replicates of transfected leaves were analyzed using the laser scanning confocal imaging system (TCS SP8, Leica, Wetzlar, Germany).

### 2.8. Analysis of DNA Sequence and Yeast One-Hybrid Analysis

The PLANTCARE (http://bioinformatics.psb.ugent.be/webtools/plantcare/html) and MatInspector (http://www.genomatix.de/online_help/help_matinspector/matinspector_help.html) software were used to analyze the identification and characterization of putative *cis*-acting elements of the *FaMADS1* promoter.

Yeast one-hybrid assay was performed using the Matchmaker^®^ Gold Yeast One-Hybrid Library Screening System (Clontech, Mountain View, CA, USA). The promoters of *FaMADS1* (*FaMADS1*-pro1 and *FaMADS1*-pro2) were constructed into the pAbAi vector. *FaMADS1*-pro1 and *FaMADS1*-pro2 lack auto-activation activities, so they were chosen for interaction tests. The test was conducted according to the instructions of Wei et al. [41].

### 2.9. Dual-Luciferase Assay

The dual-luciferase assay was performed according to Min et al. [42] and Wei et al. [41]. The vectors pGreen II 0029 62-SK (SK) and pGreen II 0800-LUC were used for building fusion vectors with FaABI5-5, TRAB1, ABI5, and FaMADS1. The proteins were expressed in tobacco (*Nicotiana benthamiana*) by agrobacterium transformation of *Agrobacterium tumefaciens* GV3101. The activities of the enzymes (FLUC and RLUC) were analyzed using the Dual-Luciferase Reporter Assay System (Promega, Madison, WI, USA, E1910) with Modulus Luminometers (Promega).

### 2.10. Statistical Assay

All determinations were conducted in triplicate, and the data are presented as means ± standard deviations. All data were analyzed for significance using SPSS software (*p* < 0.05, significance) and Origin 9.0. The error bars represent the standard deviation (SD, *n* = 3). Asterisks indicate values significantly different from the control samples (* *p* < 0.05; ** *p* < 0.01) through the Student’s *t* test.

## 3. Results

### 3.1. ABA Induced the Ripening of Strawberry Fruit

**Figure 1:**
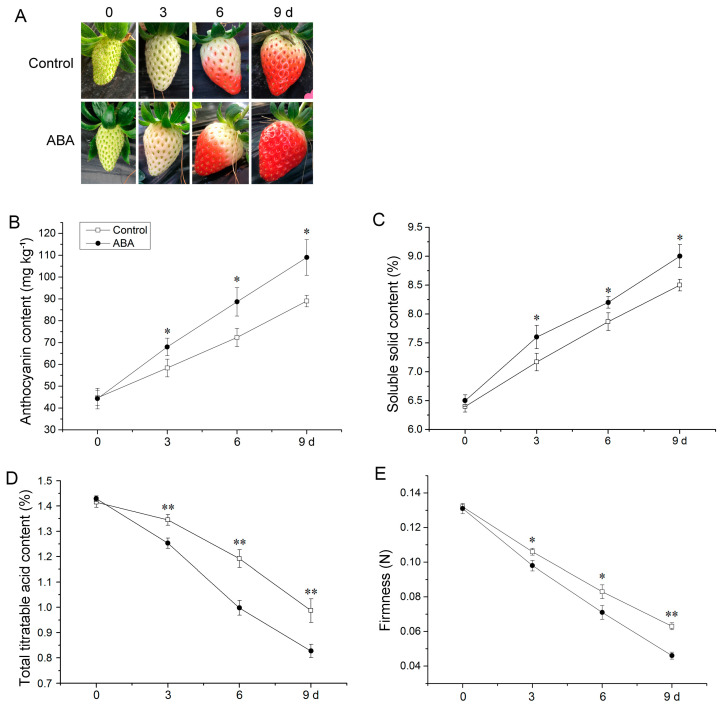
Influence of ABA on strawberry fruit phenotype (**A**), anthocyanin (**B**), soluble solid (**C**), total titratable acid (**D**), and firmness (**E**). Error bars represent standard deviation (SD, *n* = 3). Asterisks indicate values significantly different by Student’s *t* test from the control samples (* *p* < 0.05; ** *p* < 0.01).

ABA induced fruit ripening by shortening the time to attain the full red stage (Figure 1A). Anthocyanin and soluble solid accumulated rapidly with significant differences between the control and ABA-treated fruit (Figure 1B,C). However, ABA treatment reduced total titratable acid content and fruit firmness (Figure 1D,E). These results indicated that ABA stimulated the ripening of strawberry fruit.

### 3.2. Down-Regulation of FaMADS1 and Up-Regulation of Ripening-Related Gene Expressions by ABA

**Figure 2:**
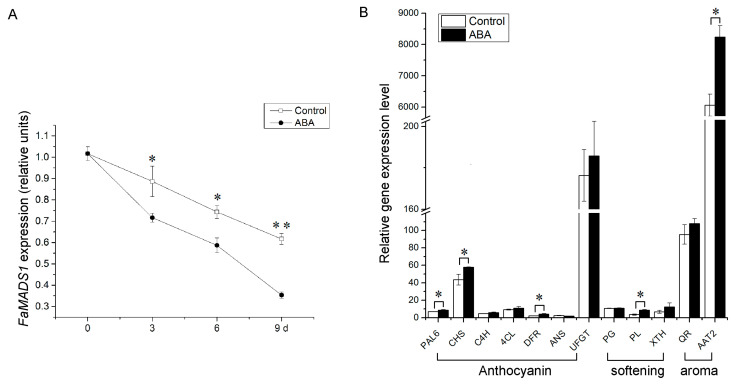
Relative expression levels of *FaMADS1* (**A**) in strawberry fruit at 0, 3, 6, and 9 d after ABA treatment and other ripening-related genes (**B**) in strawberry fruit at 3 d after ABA treatment. Anthocyanin-related genes: *FaANS*, anthocyanidin synthase; *Fa4CL*, ρ-coumarate ligase; *FaC4H*, cinnamicacid-4-hydroxylase; *FaCHS*, chalcone synthase; *FaDFR*, dihydroflavonol 4-reductase; *FaPAL6*, phenylalanine ammonia lyase; *FaUFGT*, flavonoid 3-O-glucosyltransferase. Softening-related genes: *FaPG*, Polygalacturonase; *FaPL*, pectate lyase; *FaXTH*, xyloglucan endotransglycosylase/hydrolase. Aroma-related genes: *FaAAT2*, alcohol acyltransferase; *FaQR*, quinone oxidoreductase. Error bars represent standard deviation (SD, *n* = 3). Asterisks indicate values significantly different by Student’s *t* test from the control samples (* *p* < 0.05; ** *p* < 0.01).

ABA significantly reduced the expression of *FaMADS1* and a rapid decrease trend was observed during strawberry ripening (Figure 2A). Furthermore, there was no significant difference between the control and ABA-treated fruit on the expression levels of *FaC4H*, *Fa4CL*, *FaANS*, *FaUFGT*, *FaPG*, and *FaQR*, whereas the expression of several anthocyanin biosynthesis genes (*PAL6*, *CHS*, and *DFR*), softening-related gene (*PL*), and aroma biosynthesis gene (*AAT2*) were significantly up-regulated by ABA treatment (Figure 2B).

### 3.3. Down-Regulation of FaMADS1 in FaMADS1-RNAi Strawberry Fruit

**Figure 3:**
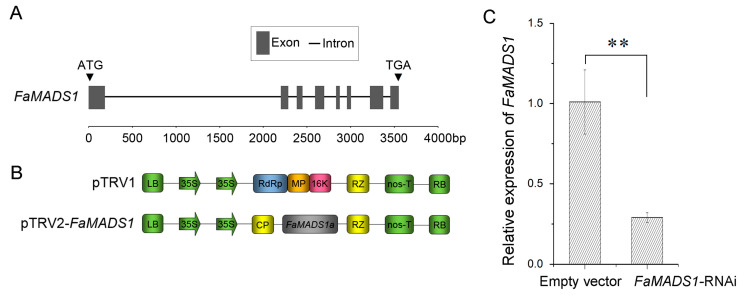
Construction of *FaMADS1*-RNAi strawberry fruit. Genomic DNA sequence analysis of FaMADS1 (**A**). Dark gray boxes represent exons and lines indicate introns. The scale indicates the length of the sequence. Schematic representation of the pTRV1 and pTRV2-*FaMADS1* VIGS constructs (**B**). CP: viral coat protein; RdRp: RNA-dependent RNA polymerase; MP: movement protein; 16 K: 16 kDa protein; RZ: ribozyme. Relative *FaMADS1* transcript levels in TRV (empty vector) and TRV-*FaMADS1* (*FaMADS1*-RNAi) strawberry fruit were measured using qRT-PCR at 7 d after infiltration (**C**). Error bars represent standard deviation (SD, *n* = 3). Asterisks indicate values significantly different by Student’s *t* test from the control samples (** *p* < 0.01).

In this study, the fruit at the DG stage was injected with a suspension of *A. tumefaciens* cells carrying a construct of *FaMADS1*-RNAi (Figure 3A,B). *FaMASD1*-RNAi decreased the *FaMADS1* transcript level by more than three folds, suggesting that the transient RNAi technique successfully manipulated *FaMADS1* expression in the strawberry fruit (Figure 3C).

### 3.4. Promoted Ripening in FaMADS1-RNAi Strawberry

**Figure 4:**
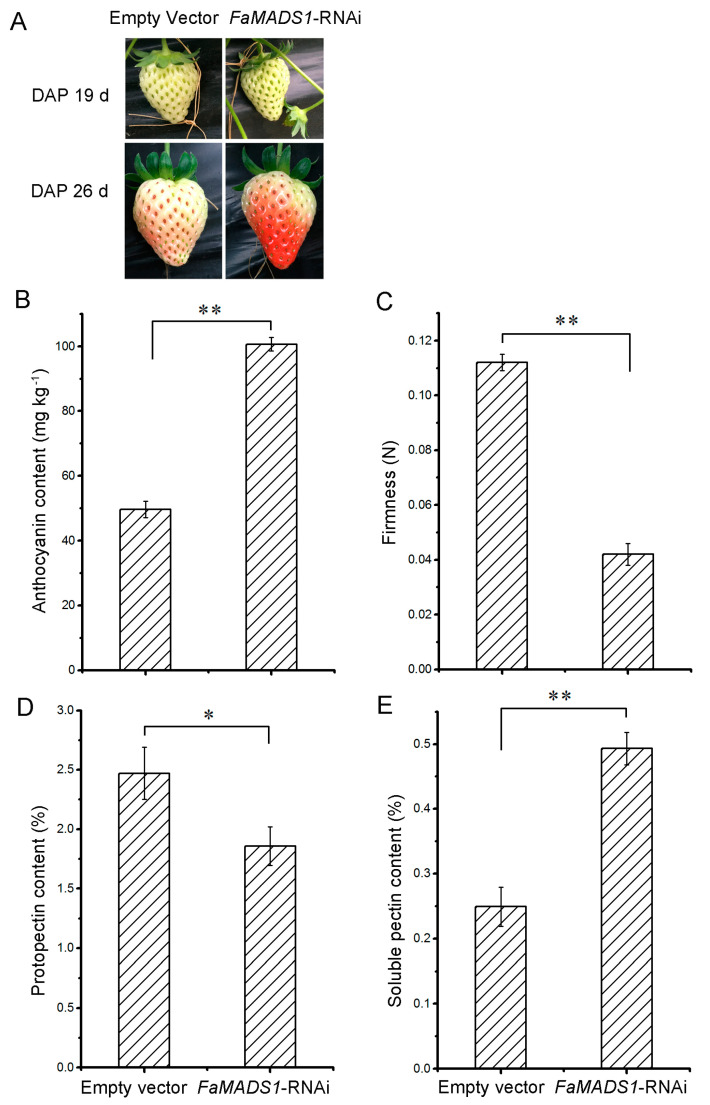
Fruit properties of *FaMADS1*-RNAi strawberry. RNAi fruit was already red when the control fruit was white at 7 d after injection (**A**). Anthocyanin content (**B**), firmness (**C**), protopectin (**D**), and soluble pectin content (**E**) were detected in control (empty vector) and *FaMADS1*-RNAi strawberry fruit at 7 d after injection. Error bars represent standard deviation (SD, *n* = 3). Asterisks indicate values significantly different by Student’s *t* test from the control samples (* *p* < 0.05; ** *p* < 0.01).

As shown in Figure 4A, *FaMASD1*-RNAi greatly promoted the ripening progression of strawberry fruit. The fruit on *FaMADS1*-RNAi plants became partially red (nearly 75%), whereas the control fruit remained in the white stage at 7 d after injection. *FaMADS1*-RNAi fruit displayed a substantial increase in anthocyanin content and a decrease in firmness (Figure 4B,C). Furthermore, *FaMADS1*-RNAi led to a decrease in protopectin and an increase in soluble pectin (Figure 4D,E).

Furthermore, *FaMADS1*-RNAi increased the levels of esters (methyl hexanoate; 1-methyl hexanoate; methyl 2-octynoate; octyl butyrate; octyl 3-methylbutyrate; (3,7,11-trimethyldodeca-1,6,10-trien-3-yl) formate; 3,7,11-trimethyl-1,6,10-dodecatrien-3-olacetate and 5-octyloxolan-2-one), ketones [DMMF and 1-(2,6,6-trimethyl-1-cyclohexen-1-yl)-1-penten-3-one], acids (octanoic acid and 3-hydroxydodecanoic acid), terpenes (2,3-dihydro-1*H*-indene), and benzene [(4aS-*cis*)-2,4a,5,6,7,8,9,9a-octahydro-3,5,5-trimethyl-9-methylene-1*H*-Benzocycloheptene]. Meanwhile, we observed increased levels of some alcohol, aldehyde, and olefin in *FaMADS1*-RNAi strawberry fruit (Table 1). This increase in aroma indicated that a metabolic boost facilitated fruit ripening in *FaMADS1*-RNAi lines.

### 3.5. Increased Ripening Gene Expressions and Enzyme Activity in FaMADS1-RNAi Strawberry Fruit

**Figure 5:**
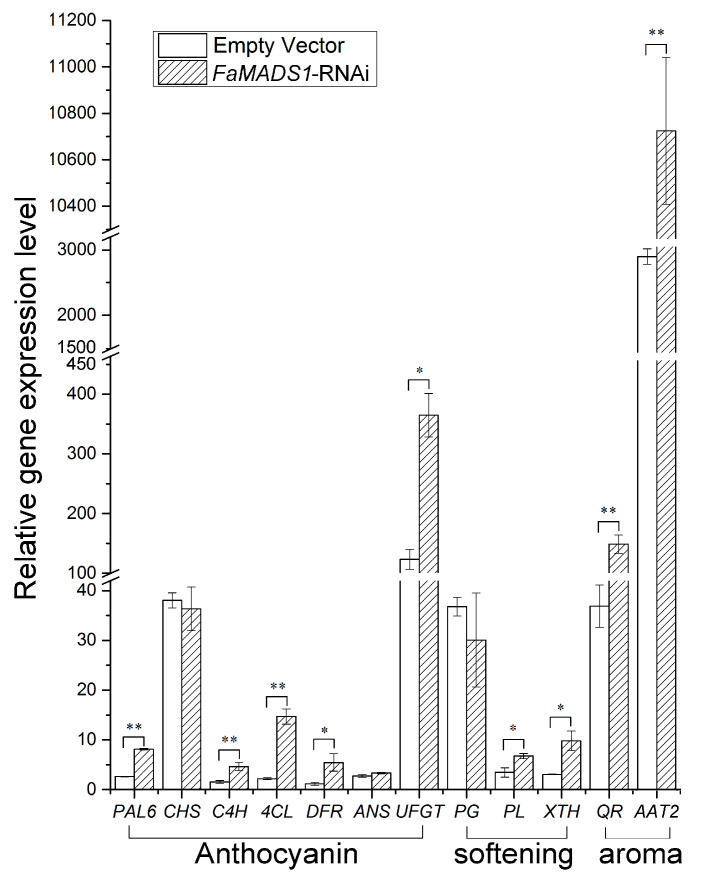
Relative expression of ripening-related genes in the control fruit (empty vector) and *FaMADS1*-RNAi fruit at 7 d after injection. Each value represents the mean of six independent fruit. Anthocyanin-related genes: *FaANS*, anthocyanidin synthase; *Fa4CL*, ρ-coumarate ligase; *FaC4H*, cinnamicacid-4-hydroxylase; *FaCHS*, chalcone synthase; *FaDFR*, dihydroflavonol 4-reductase; *FaPAL6*, phenylalanine ammonia lyase; *FaUFGT*, flavonoid 3-O-glucosyltransferase. Softening-related genes: *FaPG*, Polygalacturonase; *FaPL*, pectate lyase; *FaXTH*, xyloglucan endotransglycosylase/hydrolase. Aroma-related genes: FaAAT2, alcohol acyltransferase; *FaQR*, quinone oxidoreductase. Error bars represent standard deviation (SD, *n* = 3). Asterisks indicate values significantly different by Student’s *t* test from the control samples (* *p* < 0.05; ** *p* < 0.01).

The expression of genes related to fruit color, firmness, and aroma metabolism was significantly regulated by *FaMADS1*-RNAi (Figure 5). *FaMADS1*-RNAi greatly promoted the expression levels of *FaPAL6*, *FaC4H*, *Fa4CL*, *FaDFR*, and *FaUFGT*, which are involved in anthocyanin biosynthesis [43]. *FaMADS1*-RNAi also induced the transcription of fruit softening-related genes, such as *FaPL* and *FaXTH* [44]. Notably, the expression level of *FaQR* in *FaMADS1*-RNAi fruit was 4-fold higher than the controls, and *FaMADS1*-RNAi led to an almost 3-fold increase in the expression level of *FaAAT2*.

**Figure 6:**
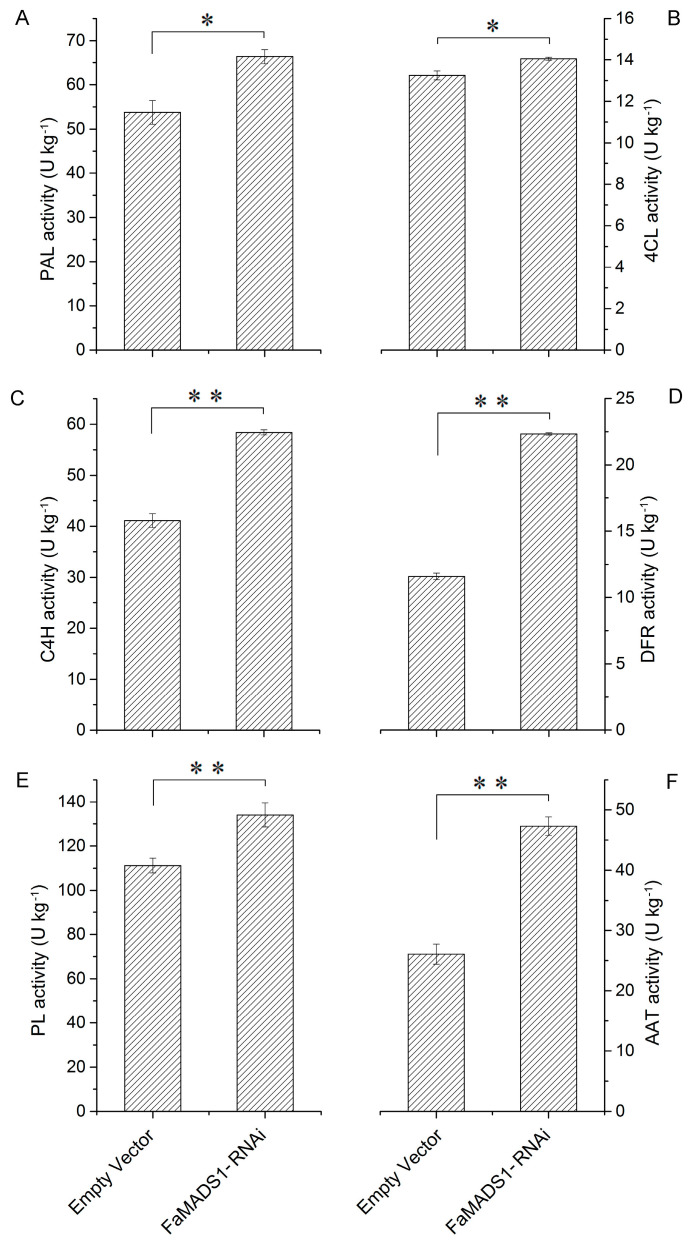
PAL (**A**), 4CL (**B**), C4H (**C**), DFR (**D**), PL (**E**), and AAT (**F**) activities in the control (empty vector) and FaMADS1-RNAi fruit at 7 d after injection. PAL, phenylalanine ammonia lyase; 4CL, ρ-coumarate ligase; C4H, cinnamicacid-4-hydroxylase; DFR, dihydroflavonol-4-reductase; PL, pectate lyase; AAT, alcohol acyltransferase. Error bars represent standard deviation (SD, *n* = 3). Asterisks indicate values significantly different by Student’s *t* test from the control samples (* *p* < 0.05; ** *p* < 0.01).

*FaMADS1*-RNAi significantly promoted the activities of PAL, 4CL, C4H, and DFR (Figure 6A–D), which are involved in the phenylpropanoid pathway of anthocyanin biosynthesis [45]. There was no significant difference in PG activity between the control and *FaMADS1*-RNAi fruit, but there was a significant increase in PL activity in *FaMADS1*-RNAi fruit (Figure 6E). Furthermore, the activity of AAT in *FaMADS1*-RNAi fruit was higher than that in the control group (Figure 6F).

### 3.6. Localization and Interaction of FaMADS1 with ABI5-5, TRAB1, and ABI5

**Figure 7:**
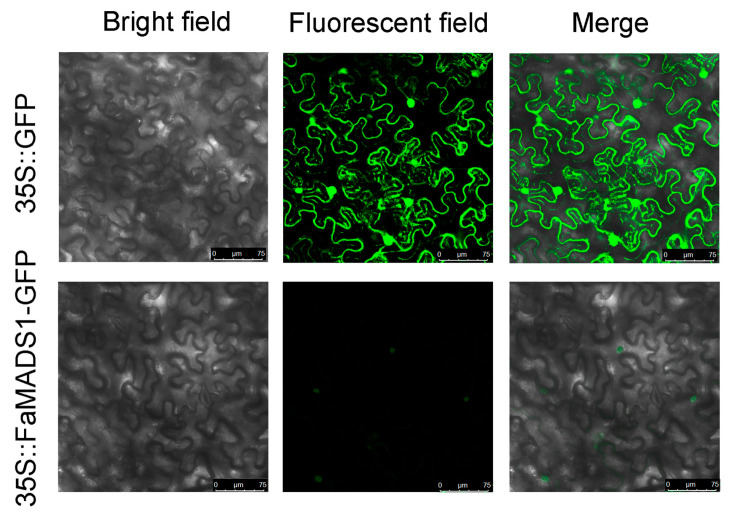
Sub-cellular localization of 35S::FaMADS1-GFP fusion protein in leaf epidermal cells of tobacco. Plasmids of 35S::GFP and 35S::FaMADS1-GFP were transformed into leaf epidermal cells by agro-infiltration, respectively. Bright field images, fluorescent field images, and the merged images of leaf epidermal cells expressing 35S::GFP or 35S::FaMADS1-GFP fusion protein.

The coding sequence of *FaMADS1* without stop codes was constructed in pBI221. The leaf epidermal cells with 35S::GFP demonstrated green fluorescence throughout the cell. By contrast, green fluorescence was especially detected in the nuclei of leaf epidermal cells expressing the 35S::FaMADS1-GFP fusion plasmids (Figure 7), suggesting that FaMADS1 was a nuclear protein.

**Figure 8:**
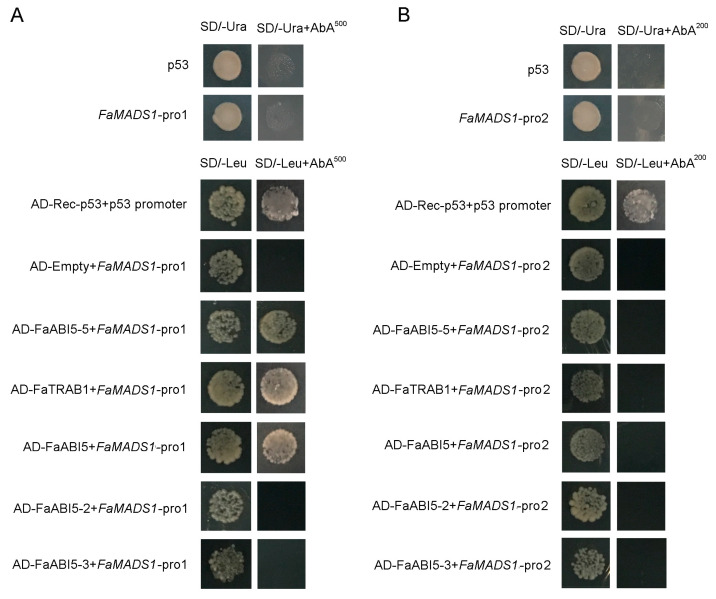
Yeast one-hybrid analysis of ABI5-5, TRAB1, ABI5, ABI5-2, and ABI5-3 binding to the *FaMADS1* promoter. Auto-activation tests of p53, *FaMADS1*-pro1, and *FaMADS1*-pro2 were experimented on SD medium lacking Ura in the presence of aureobasidin A. Interaction tests between FaMADS1 promoters with transcription factors were carried out on SD medium lacking Leu in the presence of aureobasidin A. (**A**) The interaction between ABI5-5, TRAB1, ABI5, ABI5-2, ABI5-3, and *FaMADS1*-pro1. (**B**) The interaction between ABI5-5, TRAB1, ABI5, ABI5-2, ABI5-3, and *FaMADS1*-pro2.

An online informatics survey was implemented to analyze a fragment of the *FaMADS1* promoter sequence (1095 bp upstream from the ATG start codon). A putative domain (ABRE) for responding to ABA was found (Table S2). Therefore, two sequences (*FaMADS1*-pro1 and *FaMADS1*-pro2) of *FaMADS1* promoter were cloned to study the interaction between FaMADS1 and ABA-related transcription factors (FaABI5-5, FaTRAB1, FaABI5, FaABI5-2, and FaABI5-3). FaABI5-5, FaTRAB1, and FaABI5 interacted with *FaMADS1*-pro1, whereas ABI5-2 and ABI5-3 did not interact with *FaMADS1*-pro1 (Figure 8A). FaABI5-5, FaTRAB1, FaABI5, FaABI5-2, and FaABI5-3 also did not interact with *FaMADS1*-pro2 (Figure 8B). These results revealed that *FaMADS1*-pro1 contained the active cis-acting elements, which could interact with FaABI5-5, FaTRAB1, and FaABI5.

**Figure 9:**
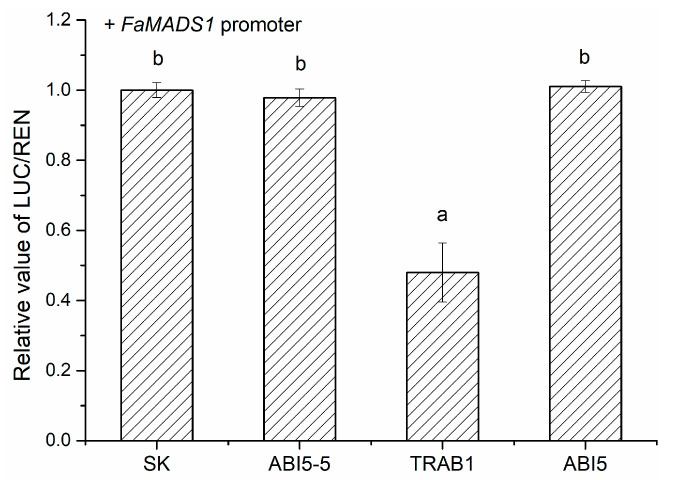
Regulatory effects of FaABI5-5, FaTRAB1, and FaABI5 on the promoters of the *FaMADS1* gene using the dual-luciferase assay. The LUC/REN ratio of the empty vector (SK) plus promoter was set as 1. Different letters above the points indicate significantly different values (*p* < 0.05) through the LSD test.

To further verify the regulatory roles of FaABI5-5, FaTRAB1, and FaABI5 in *FaMADS1*, a dual-luciferase assay was performed in tobacco leaves (Figure 9). FaTRAB1 could significantly inhibit the promoter of *FaMADS1*, while FaABI5-5 and FaABI5 had little influence on the *FaMADS1* promoter.

## 4. Conclusions and Discussion

In this study, ABA significantly promoted the accumulation of anthocyanin and soluble solid in strawberry fruit, but reduced fruit firmness and total titratable acid; thus, ABA regulated almost the entire fruit ripening process [46]. This significant acceleration of fruit ripening by ABA was accompanied by increased expression levels of series genes involved in anthocyanin biosynthesis (*PAL*, *CHS*, and *DFR*), softening (*PL*), and easier biosynthesis (*AAT*). PAL was discovered to trigger the production of anthocyanin [47]. CHS was indicated to increase the attractive red color of the strawberry fruit [48], and DFR was one of the key anthocyanin structural enzymes [49]. PL was responsible for the eliminative cleavage of pectate, and then oligosaccharides were produced with 4-deoxy-α-D-mann-4-enuronosyl groups at their non-reducing ends, resulting in fruit softening [50]. During ripening, strawberry fruit produced lots of secondary metabolites contributing to flavor and organoleptic peculiarities. Zabetakis and Holden [51] discovered more than 350 compounds from strawberries, including alcohols, esters, aldehydes, furanone derivative ketones, sulfur compounds, and terpenes; however, there were only 20 compounds actually contributing to the aroma and flavor of strawberries [52]. Alcohol acyltransferase (AAT) was involved in the biosynthesis of short-chain esters, contributing to the blend of volatiles and defining the fruit aroma [53]. These results demonstrated that ABA regulated ripening by promoting softening metabolism and biosynthesis of anthocyanin and aroma in strawberries.

Studies have suggested that some MADS members contributed to the fruit ripening of tomatoes [54], grape [55], strawberries [56,57], and bananas [58]. The MADS-box gene *FaMADS1* was suppressed with an accelerated ripening process following ABA treatment [28]. These results confirmed that *FaMADS1* might play a negative role in fruit ripening via the downstream ABA signal transduction pathway connected to related metabolic processes.

Previous studies have reported that tobacco rattle virus (TRV)-mediated VIGS was a rapid and feasible technique for analyzing fruit ripening of tomato [59] and strawberry [60,61]. In the present study, the negative role of *FaMADS1* in the ripening of strawberry fruit was verified through a gene silencing technique. Compared with the control (empty vector), *FaMADS1*-RNAi fruit exhibited accelerated ripening with high levels of anthocyanin and soluble pectin, low firmness, and protopectin content. In addition, an increase in the content levels of ester, ketone, acid, terpenes, and benzene was observed in the *FaMADS1*-RNAi fruit. The down-regulation of *FaMADS1* resulted in increased expression levels of anthocyanin-related genes (*PAL6*, *C4H*, *4CL*, *DFR*, and *UFGT*), softening-related genes (*PL* and *XTH*), and aroma-related genes (*QR* and *AAT2*). In tomato, the silence experiment also caused changes in fruit ripening. Mekontso et al. [62] found that postharvest ripening could be delayed by knocking down miR160a, and some related genes, such as *ARF10A*, *ARF10B*, and *ZRF17*, were up-regulated. Meanwhile, *FaMADS1*-RNAi significantly promoted the activities of PAL, 4CL, C4H, DFR, PL, and AAT enzymes. These results collectively suggested that the *FaMADS1* gene had a strong negative function in strawberry ripening.

The gene *ABI* encoded protein PP2C in the ABA signal pathway. *FaABI1* and *FaABI2* negatively regulated strawberry fruit ripening, whereas *FaABI4* and *FaABI5* positively regulated fruit ripening [5,63]. Previous studies have confirmed that ABI and TRAB are the major transcription factors in the ABA signaling pathway [18,63,64,65,66]. In the present study, yeast one-hybrid analysis revealed that FaABI5-5, FaTRAB1, and FaABI5 could interact with *FaMADS1*-pro1. Furthermore, the dual-luciferase assay confirmed that the promoter of *FaMADS1* could be significantly inhibited by FaTRAB1, whereas FaABI5-5 and FaABI5 had little influence on the promoter of *FaMADS1*. These results suggested that the ABA signal was transmitted to the *FaMADS1* promoter with the interaction of FaTRAB1. *FaMADS1* regulated the transcriptional level of the downstream anthocyanin-related genes, softening-related genes, and aroma-related genes, eventually initiating fruit ripening.

**Figure 10:**
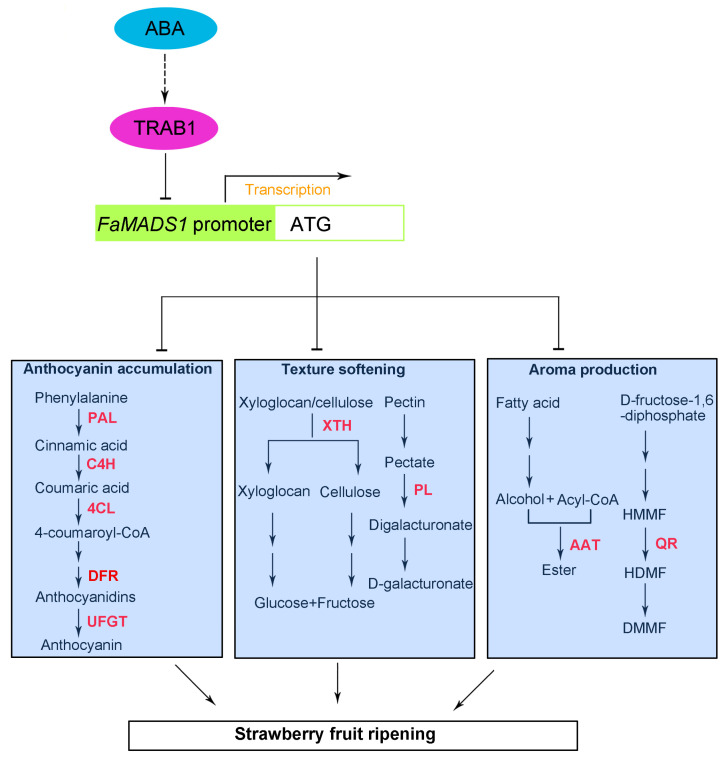
A model for ABA receptors-mediated signaling pathway connected to *FaMADS1* regulating strawberry fruit ripening. In the presence of ABA, ABA signal activated ABI and TRAB transcription factors, and then *FaMADS1* was inhibited. Depression of *FaMADS1* induced the expression of anthocyanin-related *FaPAL*, *FaC4H*, *Fa4CL*, and *FaUFGT*, softening-related *FaPL* and *FaXTH*, and aroma-related *FaQR* and *FaAAT*. Abbreviations: AAT2, alcohol acyltransferase; ABA, abscisic acid; 4CL, ρ-coumarate ligase; C4H, cinnamicacid-4-hydroxylase; PAL6, phenylalanine ammonia lyase; PL, pectate lyase; QR, quinone oxidoreductase; SnRK2s, phosphorylation of ABA-activated sucrose non-fermenting 1-related protein kinases subfamily 2; TRAB, transcription factor responsible for ABA regulation; UFGT, flavonoid 3-O-glucosyltransferase; XTH, xyloglucan endotransglycosylase/hydrolase. The solid arrows indicate direct action and the dashed arrow indicates indirect action.

In conclusion, *FaMADS1* expression negatively depended on ABA to regulate strawberry fruit ripening. The yeast one-hybrid analysis and dual-luciferase assay suggested that the *FaMADS1* promoter was inhibited by the interaction with FaTRAB1, resulting in the blockage of *FaMADS1* translation. ABA application and *FaMADS1*-RNAi data reflected a negative regulation of *FaMADS1* to the downstream anthocyanin-related genes (*PAL6*, *C4H*, *4CL*, *DFR*, and *UFGT*), softening-related genes (*PL* and *XTH*), and aroma-related genes (*QR* and *AAT2*), as well as the enzyme activities (Figure 10). This observation enhanced our understanding of the molecular mechanism of ABA signaling during the non-climacteric fruit ripening.

## Figures and Tables

**Table 1:** Main aroma compounds in *FaMADS1*-RNAi strawberry fruit.

Compounds (μg kg^−1^ FW)	Empty Vector	*FaMADS1*-RNAi
**Esters**		
Methyl hexanoate	0.424 ± 0.101	1.021 ± 0.132 *
1-Methyl hexanoate	0.000 ± 0.000	0.065 ± 0.018 **
Methyl 2-octynoate	0.000 ± 0.000	0.083 ± 0.008 **
Octyl butyrate	0.000 ± 0.000	0.129 ± 0.001 **
Octyl 3-methylbutyrate	0.000 ± 0.000	0.377 ± 0.027 **
(3,7,11-Trimethyldodeca-1,6,10-trien-3-yl) formate	0.000 ± 0.000	0.286 ± 0.128 **
3,7,11-Trimethyl-1,6,10-dodecatrien-3-olacetate	0.000 ± 0.000	0.184 ± 0.044 **
**Ketones**		
5-Octyloxolan-2-one	0.000 ± 0.000	0.608 ± 0.291 **
Methyl *n*-hexyl ketone	52.400 ± 0.000	52.400 ± 0.000
4-Methoxy-2,5-dimethylfuran-3-one (DMMF)	0.000 ± 0.000	0.336 ± 0.058 **
1-(2,6,6-Trimethyl-1-cyclohexen-1-yl)-1-penten-3-one	0.000 ± 0.000	0.352 ± 0.007 **
**Alcohols**		
Octan-2-ol	0.332 ± 0.027	0.000 ± 0.000 **
3,7-Dimethyl-1,6-octadien-3-ol	0.957 ± 0.056	1.453 ± 0.265
2-(4-Methyl-1-cyclohex-3-enyl) propan-2-ol	0.071 ± 0.020	0.123 ± 0.002
3,7,11-Trimethyl-1,6,10-Dodecatrien-3-ol	1.569 ± 0.038	14.913 ± 4.932
**Acids**		
7-Oxooctanoic acid	0.090 ± 0.019	0.155 ± 0.034
Octanoic acid	0.000 ± 0.000	0.073 ± 0.039 **
3-Hydroxydodecanoic acid	0.000 ± 0.000	0.044 ± 0.005 **
**Aldehydes**		
€-hex-2-enal	1.971 ± 0.049	0.000 ± 0.000 **
Nonanal	0.203 ± 0.028	0.270 ± 0.053
Decanal	0.061 ± 0.004	0.050 ± 0.007
**Olefins**		
D-1-methyl-4-(1-methylethenyl)-cyclohexene	0.044 ± 0.000	0.061 ± 0.026
(Z)-5-Undecene	0.058 ± 0.017	0.000 ± 0.000 **
(Z)-7,11-Dimethyl-3-methylene-1,6,10-dodecatriene	0.090 ± 0.017	1.060 ± 0.153 *
1-(1,5-Dimethyl)-4-methy-benzene	0.000 ± 0.000	0.047 ± 0.027 **
1-(1,5-Dimethyl-4-hexenyl)-4-methylbenzene	0.000 ± 0.000	0.090 ± 0.016 **
2,6-Dimethyl-6-(4-methyl-3-pentenyl)bicyclo [3.1.1]hept-2-ene	0.000 ± 0.000	0.147 ± 0.027 **
(1R,4aR,8aS)-7-methyl-4-methylidene-1-propan-2-yl-2,3,4a,5,6,8a-hexahydro-1*H*-naphthalene	0.091 ± 0.029	0.000 ± 0.000 **
3,7,11-Trimethyl-1,3,6,10-dodecatetraene	0.000 ± 0.000	0.291 ± 0.068 **
7-epi-*cis*-sesquisabinene hydrate	0.000 ± 0.000	0.229 ± 0.105 **
**Terpenes**		
2,3-Dihydro-1*H*-indene	0.000 ± 0.000	0.052 ± 0.004 **
**Benzenes**		
Butylbenzene	0.127 ± 0.032	0.151 ± 0.010
1-Methyl-2-propylbenzene	0.095 ± 0.012	0.142 ± 0.019
(4aS-*cis*)-2,4a,5,6,7,8,9,9a-Octahydro-3,5,5-trimethyl-9-methylene-1*H*-Benzocycloheptene	0.000 ± 0.000	0.037 ± 0.000 **
**Others**		
1-Methyl-6,7-dioxabicyclo[3.2.1]octane	0.093 ± 0.001	0.000 ± 0.000 **
2,4-bis(1,1-dimethylethyl)-phenol	0.182 ± 0.029	0.000 ± 0.000 **
1-Bromo-3,7,11-trimethyl-2,6,10-dodecatriene	0.000 ± 0.000	0.086 ± 0.018 **

Note: Error bars represent standard deviation (SD, *n* = 3). Asterisks indicate values significantly different by Student’s *t* test from the control samples (* *p* < 0.05; ** *p* < 0.01).

## Data Availability

Not applicable.

## Supplementary Materials

The following supporting information can be downloaded at: https://www.mdpi.com/article/10.3390/foods12091802/s1, Table S1: All primers used in the study for PCR analysis; Table S2: Bioinformatics analysis of the *FaMADS1* gene promoter.
