# Supplementary material for: Participation of FaTRAB1 Transcription Factor in the Regulation of FaMADS1 Involved in ABA-Dependent Ripening of Strawberry Fruit
Source: Foods. 2023 Apr 26;12(9):1802. doi: 10.3390/foods12091802 (PMC10177999; doi:10.3390/foods12091802)
Supplement: Supplementary file 1 [file foods-12-01802-s001.zip › foods-2323416-supplementary.pdf]

Table S1. All primers used in the study for PCR analysis.

gene name	Forward primers (5´-3´)	Reverse primers (5´-3´)	Accession
FaMADS1 -Q	TTACAATTAAGGAAATGGGGA G	GCTGCATGCTATCTCAATTATCA	
FaMADS1 -RT	AACTTTAGACAAACTTGGGAA C	GGCTGGTGTGGCTGTAGGCAT	
FaMADS1 -RNAi	TGCTCTAGAATGGGGAGGGGA AGAGTGG	CCGGAATTCTCAGAGCATCCAACC AGG	
FaMADS1 -GFP	GAATTCATGGGGAGGGGAAGA GTGG	GTCGACGAGCATCCAACCAGG	
FaMADS1 -pro1	CGAGCTCATGAGACTTTAATTC TACAC	CCGCTCGAGAATCCACGGTTACCA TTCAC	
FaMADS1 -pro2	CGAGCTCATGAGACTTTAATTC TACAC	CCGCTCGAGCTTGCGTGTCTAGGA CCTAC	
FaMADS1 -LUC	GTCGACGGTATCGATAAGCTT ATGAGACTTTAATTCTACAC	CGCTCTAGAACTAGTGGATCCAAT CCACGGTTACCATTCAC	
FaPAL6	GACAAGGTGTTCAAGGCAATC TG	AATCAAACCAATTTCAGCCAAAAG	HM641823
FaCHS	GCTGTCAAGGCCATTAAGGA	GAGCAAACAACGAGAACACG	XM_00430649 5
Fa4CL	GGAGCCAAATCATGAAAGG	GTCGATGTACCCTATATCACC	(EST)
FaC4H	TACCTTCCGTTTGGTGTCGG	GCAGGCTAAACTGTCCACCT	(EST)
FaDFR	ACGAAGTGATAAAGCCAACA	AAACACCAACCTCCGAAC	AF029685
FaANS	CGTGAGACCCAAAGAGGA	ATGCCGTGGTTGATAAGG	AY695818
FaUFGT	ACGAAGTGATAAAGCCAACA	TATGAGCACCGAACCAAAA	AY575056
FaPG1	TCGGAAGTCTCGGAAGATACG ACA	CGAGGCGGTGGTAGCTTTAGGA	AY282613
FaPL	TGACTCCCTTGCTGCTTCTT	TCTACTGCGTGCTCATTCCA	EF441273
FaXTH	ACTCTGCTCTTGAGCATAGTGC	GAGCTGAATCTCATTGCCACC	(EST, Opazo et al., 2010)
FaQR	CCTTGGGTGCTGCTGATTTG	GTGGCGTTGCTGGACCTACTATT	AY048861

FaAAT2	GGAGGACATCATGGATTGGAG TTGC	GGGGATCTTGTTCTAGCATAGCC	JN089766
FaABI5-5	ATGGGTGCCAATATGAATTT	TTACCATAGGACCGGTCT	
FaABI5-5-dan	CCCGGGTATGGGTGCCAATAT GAATTT	CCGCTCGAGTTACCATAGGACCGG TCT	
FaABI5-5- SK	GCGGCCGCTCTAGAACTAGTG ATGGGTGCCAATATGAAT	GGTCGACGGTATCGATATAGC TTACCATAGGACCGGTCT	
FaTRAB1	ATGCACAAGCTTTTCAGAACA T	TTAGTAGGCCTAGAATGGAA	
FaTRAB1-dan	CCCGGGTATGCACAAGCTTTTC AGAACAT	CCGCTCGAGTTAGTAGGCCTAGAA TGGAA	
FaTRAB1- SK	GCGGCCGCTCTAGAACTAGTG ATGCACAAGCTTTTCAGA	GGTCGACGGTATCGATATAGCTTA GTTAGGCCTAGATGG	
FaABI5	ATGATTAGCTCGTATACTGG	TCACACAGCACAACTGTGACTC	
FaABI5-dan	CCCGGGTATGATTAGCTCGTAT ACTGG	CGAGCTCTCACACAGCACAACTGT GACTC	
FaABI5- SK	GCGGCCGCTCTAGAACTAGTG ATGATTAGCTCGTATACT	GGTCGACGGTATCGATATAGCTCA CACAGCACAACTGTG	
FaABI5-2	ATGGGGATACAGACAATGGG	TCAGAATGGAGCTGATGTG	
FaABI5-2-dan	CCCGGGTATGGGGATACAGAC AATGGG	CGAGCTCTCAGAATGGAGCTGATG TG	
FaABI5-3	ATGGATGATAGAACTTTGG	TTAAAAGGAAGCTGAACTGG	
FaABI5-3-dan	CCCGGGTATGGATGATAGAAC TTTGG	CGAGCTCTTAAAAGGAAGCTGAAC TGG	
FaActin	TGGGTTTGCTGGAGATGAT	CAGTAGGAGAACTGGGTGC	AB116565

Table S2. Bioinformatic analysis of the *FaMADS1* gene promoter.

Element	Description	Sequence	Position
ABRE (ABA response element)	ABA inducible transcriptional activator	ACGTG	-1095 (+)

Note: The position of the indicated *cis*-acting element was expressed relative to ATG codon.
